# Microbial Diversity in Actively Forming Iron Oxides from Weathered Banded Iron Formation Systems

**DOI:** 10.1264/jsme2.ME18019

**Published:** 2018-11-16

**Authors:** Emma J. Gagen, Alan Levett, Jeremiah Shuster, Danielle Fortin, Paulo M. Vasconcelos, Gordon Southam

**Affiliations:** 1 School of Earth and Environmental Sciences, The University of Queensland St Lucia, QLD 4072 Australia; 2 School of Biological Sciences, The University of Adelaide Adelaide, SA, 5005 Australia; 3 CSIRO Land and Water, Contaminant Chemistry and Ecotoxicology PMB2, Glen Osmond, SA 5064 Australia; 4 Department of Earth and Environmental Sciences, The University of Ottawa ON K1N 6N5 Canada

**Keywords:** canga, iron duricrust, Karijini, *Leptothrix*, *Anaeromyxobacter*

## Abstract

The surface crust that caps highly weathered banded iron formations (BIFs) supports a unique ecosystem that is a post-mining restoration priority in iron ore areas. Geochemical evidence indicates that biological processes drive the dissolution of iron oxide minerals and contribute to the ongoing evolution of this duricrust. However, limited information is available on present-day biogeochemical processes in these systems, particularly those that contribute to the precipitation of iron oxides and, thus, the cementation and stabilization of duricrusts. Freshly formed iron precipitates in water bodies perched on cangas in Karijini National Park, Western Australia, were sampled for microscopic and molecular analyses to understand currently active microbial contributions to iron precipitation in these areas. Microscopy revealed sheaths and stalks associated with iron-oxidizing bacteria. The iron-oxidizing lineages *Sphaerotilus*, *Sideroxydans*, and *Pedomicrobium* were identified in various samples and *Leptothrix* was common in four out of five samples. The iron-reducing bacteria *Anaeromyxobacter dehalogens* and *Geobacter lovleyi* were identified in the same four samples, with various heterotrophs and diverse cyanobacteria. Given this arid, deeply weathered environment, the driver of contemporary iron cycling in Karijini National Park appears to be iron-reducing bacteria, which may exist in anaerobic niches through associations with aerobic heterotrophs. Overall oxidizing conditions and *Leptothrix* iron-oxidizers contribute to net iron oxide precipitation in our sampes, rather than a closed biogeochemical cycle, which would result in net iron oxide dissolution as has been suggested for canga caves in Brazil. Enhancements in microbial iron oxide dissolution and subsequent reprecipitation have potential as a surface-crust-ecosystem remediation strategy at mine sites.

High-grade iron ore deposits that form from the weathering of banded iron formations (BIFs) are often capped by a hard, erosion-resistant duricrust known as canga (5, 9, 26, 31). Over geological time, goethite (FeO[OH]) formation within cangas results in the overall enrichment of iron content relative to BIF, *i.e.*, approximately 2- to 3-fold more iron (9). Geochemical evidence from Brazilian cangas indicates that biological processes are the drivers of iron dissolution and, thus, iron mobilization in these systems (33, 37, 38). The presence of lithified biofilms and well-preserved cellular structures via biomineralization suggests that microorganisms have also played an intimate role in iron oxide precipitation within cangas (32). Goethite dissolution and reprecipitation processes that contribute to the enrichment of iron in cangas and the evolution of this surface crust are ongoing (33, 37, 38). However, limited information is currently available on the exact mechanisms underlying biogeochemical iron cycling in cangas. More specifically, the presence, diversity, and importance of microbial groups contributing to iron dissolution and precipitation processes in canga formation remain unknown. Parker *et al.* (34) investigated biogeochemical processes in caves in the canga areas of Brazil and suggested that microbial processes contribute to overall iron oxide dissolution and cave formation. To date, no study has demonstrated how microbial processes contribute to iron oxide formation within canga ecosystems. A clearer understanding of the processes that lead to the ongoing cycling of iron and, thus, canga cementation and stabilization is fundamental for post-mining rehabilitation strategies that require the reformation of cangas. Cangas host a unique ecosystem (23–25) that is increasingly becoming a restoration priority post-mining.

The aim of the present study was to investigate the role and types of microbial processes contributing to the net formation of iron oxides in canga ecosystems. Karijini National Park, in the Hamersley region of northwest Western Australia (Fig. 1A) was selected as a study location because it offers a unique opportunity to study microbial iron cycling in a canga ecosystem under present-day arid (200–250 mm rain annum^−1^ evaporation exceeds rainfall by 7–13-fold in this region [8, 21]) conditions. The park contains a series of deep gorges that cut through several members of the Hamersley Group Archean BIF. The gorges dissect through channel iron deposits and deeply weathered BIFs (cangas) at the upper levels, whereas relatively fresh and unweathered BIFs are exposed on the floor of the gorges. Seepage along gorge walls permits the sampling of active weathering solutions from different stratigraphic levels. Moreover, Karijini National Park is completely surrounded by active or pending mining tenements (Fig. 1B) primarily for iron ore, some that include sections of the park itself (15). An understanding of the biogeochemical processes that contribute to iron oxide formation in cangas in this area may benefit the park and surrounding mines, and potentially serve as a platform for future post-mining rehabilitation projects that require the re-formation of surface crusts.

## Methods

### Field site and sample acquisition

Water samples and a number of distinct, freshly-precipitated iron oxides were collected within Karijini National Park in February 2015 under license CE004581 issued by the Department of Parks and Wildlife, Western Australia. At each sampling location, the pH of the water was measured using MColorpHast^TM^ pH strips 4.0–7.0 (Merck KGaA, Darmstadt, Germany). Water samples for chemical analyses were passed through 0.22-μm filters and stored in sterile Falcon tubes. Iron oxide samples included a mat from a small pool in Dales Gorge, S 22°28′39.7″ E 118°33′24.7″ (*e.g.* Fig. 2A, samples referred to as Dales Gorge iron mats and abbreviated as Dales pool in the Figures), a precipitate on plants growing on a wall at Dales Gorge, S 22°28′36.0″ E 118°33′10.2″ (Fig. 2B, samples abbreviated to Dales wall in the Figures), a thick iron-coated mat and small iron-coated bubbles from a pool of water perched on BIF in Knox Gorge, S 22°22′20.2″ E 118°17′59.9″ (Fig. 2C and D, samples abbreviated to Knox mats and Knox spots in Figures), and a sheen from a small pond supporting abundant aquatic vegetation in Hancock Gorge, S 22°21′32.4″ E 118°17′02.6″ (Fig. 2E, samples referred to as Hancock Gorge). Since it was not possible to obtain exact GPS coordinates within the gorges, the coordinates of sample locations were recorded on maps and cross-referenced using Google Earth satellite imagery post-sampling.

Iron mats from Dales Gorge and Knox Gorge were collected by filtering approximately 60 mL of material onto a Sterivex^TM^ 0.22-μm filter unit (EMD Millipore Corporation, Billerica MA, USA). The iron oxide film in Knox Gorge was collected with a sterile plastic pipette (together with approximately 10 mL of liquid). The iron precipitates that associated with plant material from Dales and Hancock Gorges were sampled directly into tubes using sterile spoons. Samples were snap frozen in the field using a CX100 Cryo-Exchange Dry Shipper (Taylor-Wharton, Theodore, AL, USA). Additional samples for light and electron microscopies were fixed using 2.5%_(aq)_ glutaraldehyde.

### Water chemistry

Filtered water samples were acidified to a final concentration of 7%_(aq)_ nitric acid and digested in a MARS Xpress microwave with Teflon tubes (160°C for 10 min, followed by 170°C for 10 min). Digested samples were diluted to a final concentration 5%_(aq)_ nitric acid and analyzed by inductively coupled plasma optical emission spectrophotometry (ICP-OES) using a Perkin Elmer Optima 7300DV with argon as the plasma gas at 15 L min^−1^. Samples were analyzed for soluble metals (detection limits in ppb given in parentheses): Al (1.2), As (6.3), B (1.5), Ba (0.04), Ca (0.5), Cd (0.1), Co (0.4), Cr (0.4), Cu (0.4), Fe (0.3), K (0.3), Mg (0.1), Mn (0.04), Mo (0.6), Na (0.2), Ni (0.5), P (2.9), Pb (1.7), S (0.2), Se (13), and Zn (0.2), and results are summarized in Table 1.

### DNA extraction

In samples collected on Sterivex^TM^ filters and snap frozen, filter units were cracked open in the laboratory, and the filter was removed using sterile tweezers and then placed directly into a screw cap tube for DNA extraction. In other samples, 3–5 mL of thawed sample was centrifuged at 13,000×*g* for 10 min in a screw cap tube and the supernatant was removed for DNA extraction from the harvested biomass. DNA was extracted using a bead-beating cetyltrimethylammonium bromide (CTAB)-based method coupled with the final column-based purification steps of the PowerSoil^®^ DNA isolation kit (Mo Bio Laboratories, Carlsbad, CA, USA) as outlined in Gagen *et al.* (16). Less than 30 ng of DNA extracted from each sample was used as a template in a PCR reaction to amplify the V6–V8 region of the 16S rRNA gene using the universal primers 926f and 1392r (14) adapted to contain Illumina-specific adapter sequences (adapter sequences in capitals): 926F: 5′-TCGTCGGCAGCGTCAGATGTGTATAAGA GACAGaaactyaaakgaattgacgg-3′ and 1392wR: 5′-GTCTCGTGGG CTCGGGTCTCGTGGGCTCGGAGATGTGTATAAGAGACAG acgggcggtgtgtrc-3′. Libraries were prepared as outlined by Illumina (#15044223 Rev B), except that Q5 Hot Start High-Fidelity polymerase and PCR mastermix were used (New England Biolabs, Ipswich, MA, USA). PCR amplicons were purified using Agencourt AMPure XP beads (Beckman Coulter, Brea, CA, USA). Purified DNA was indexed with unique 8-bp barcodes using the Illumina Nextera XT v2 Index Kit sets A-D (Illumina, San Diego, CA, USA) and the same PCR mastermix as previously reported. Indexed amplicons were pooled together in equimolar concentrations and sequenced on a MiSeq Sequencing System (Illumina) using paired end sequencing with V3 300 bp chemistry in accordance with the manufacturer’s protocol at the Australian Centre for Ecogenomics, The University of Queensland.

Sequences have been submitted to the National Center for Biotechnology Information (NCBI) Sequence Read Archive under BioProject number SUB3614051.

### Sequencing and sequence analyses

Sequences were processed using MOTHUR (36) as per MiSEQ SOP (https://www.mothur.org/wiki/MiSeq_SOP accessed 30^th^ September 2017) (29) with only minor changes to allow for the processing of forward reads only. The protocol used was as follows: forward reads were trimmed on quality (quality average of 35 across a sliding window of 50 nt), the primer was removed, and reads were then trimmed to 230 nt. Sequences with ambiguous bases and homopolymeric structures (8 nt repeats) were removed before alignment with the silva.nr.v132 database. Alignment positions were compressed where every position was identical in the alignment and sequences that differed by less than 2 nt were then clustered. Chimeras identified using the silva.gold.align database version 132 as a reference were removed and sequences were classified using the silva.nr.v132 database as a reference. Sequences classified as Eukaryota, Chloroplast, Mitochondria, or unknown were removed and the remaining sequences were clustered into Operational Taxonomic Units (OTUs) at a distance of ≤0.03. Singletons (OTUs that occur only once in the entire dataset) were removed from further processing. A distance matrix for all OTUs was calculated for weighted and unweighted UniFrac community structure comparisons. Shared OTUs were identified and used as the basis for diversity analyses. Libraries were subsampled to the lowest sized library (3,717 unique OTUs) before generating a heatmap to visualize shared OTUs and before calculating Yue and Clayton (47) and Jaccard (4) measures of dissimilarity. A principle coordinates analysis was performed on Jaccard distances between libraries. Correlations between geochemical data and axes on the principle coordinates analysis were examined with Pearson’s correlation coefficient. OTU associations with geochemical data were also investigated using Pearson’s correlation coefficient. In cases in which an element was below the detection limit in some samples, a value that was half of the detection limit was used for these statistical correlations to reduce bias (7). A representative sequence from each of the major OTUs was compared to publicly available sequences using the Nucleotide Basic Local Alignment Search Tool (BLASTn) (1) at the NCBI and the non-redundant nucleotide collection, excluding uncultured and environmental sample sequences.

### Light and transmission electron microscopies

Phase contrast and fluorescence light microscopies (LM) of samples that had been field fixed using 2.5%_(aq)_ glutaraldehyde were performed using a Nikon Ci-L Fluorescence Microscope with a B-2a filter for visualizing cells stained with SYTO9^®^ (Life Technologies, Eugene, Oregon, USA) as per the manufacturer’s instructions.

Samples of iron mats and mineralized iron films were prepared for transmission electron microscopy (TEM) using conventional embedding techniques modified from Graham and Beveridge (19). Fixed samples were enrobed in 2%_(wt/vol)_ Noble agar, dehydrated in sequential 25, 50, 75%_(aq)_, and 3×100% acetone, and embedded in Epon resin. Embedded samples were made into ultra-thin sections with a thickness of approximately 80 nm using a 4 Leica Ultramicrotome. Ultra-thin sections were collected on 200-square mesh copper grids and analyzed using a JEOL 1010 or JEOL 1011 Transmission Electron Microscope operating at 80 kV and a JEOL JEM 2100 LaB_6_ Transmission Electron Microscope operating at 250 kV. The latter TEM is equipped with an Oxford SDD thin-window Energy Dispersive X-Ray (EDS) detector. Spot analyses were performed on selected samples. Samples were prepared without the addition of heavy metal fixatives, and, thus, the electron densities of these ultrathin sectioned samples are the result of naturally immobilized metals.

## Results

### Water chemistry and microscopy

Soluble iron concentrations ranged from 0.08 ppm in the water dripping from the wall at Dales Gorge with iron-coated plants growing on it to 2.20 ppm at the small pool sampled nearby in Dales Gorge. At all locations, pH ranged between 5.0 and 6.0 (Table 1). Among the elements analyzed by ICP-OES, B, Ba, Ca, Co, Fe, K, Mg, Mo, Mn, Na, and S were detected in all water samples (Table 1). Mg, Ca, Na, and K measurements were within the ranges reported previously by Hedley *et al.* (21) in and around Karijini National Park, and the low levels of S detected were presumed to be present entirely as SO_4_^2−^ based on previous measurements of SO_4_^2−^ by Hedley *et al.* (21). LM and electron microscopy revealed microbial rods, cocci, and filaments intimately associated with iron oxide particles (*e.g.*, Fig. 3A and B) and the cell envelopes of many of these cells were coated in iron (Fig. 4A–D). Other cells were enclosed by larger, tens of micrometers in size diffuse assemblies of iron oxide, presumably caught in an exopolymeric substance (*e.g.*, Fig. 4C). Features interpreted as stalks produced by some neutrophilic iron oxidizers (*e.g.*, Fig. 4A and D) and empty iron-coated sheaths (Fig. 3C and D) were also observed in Dales Gorge and Knox Gorge samples.

### Microbial community structure

**Fig. 1:**
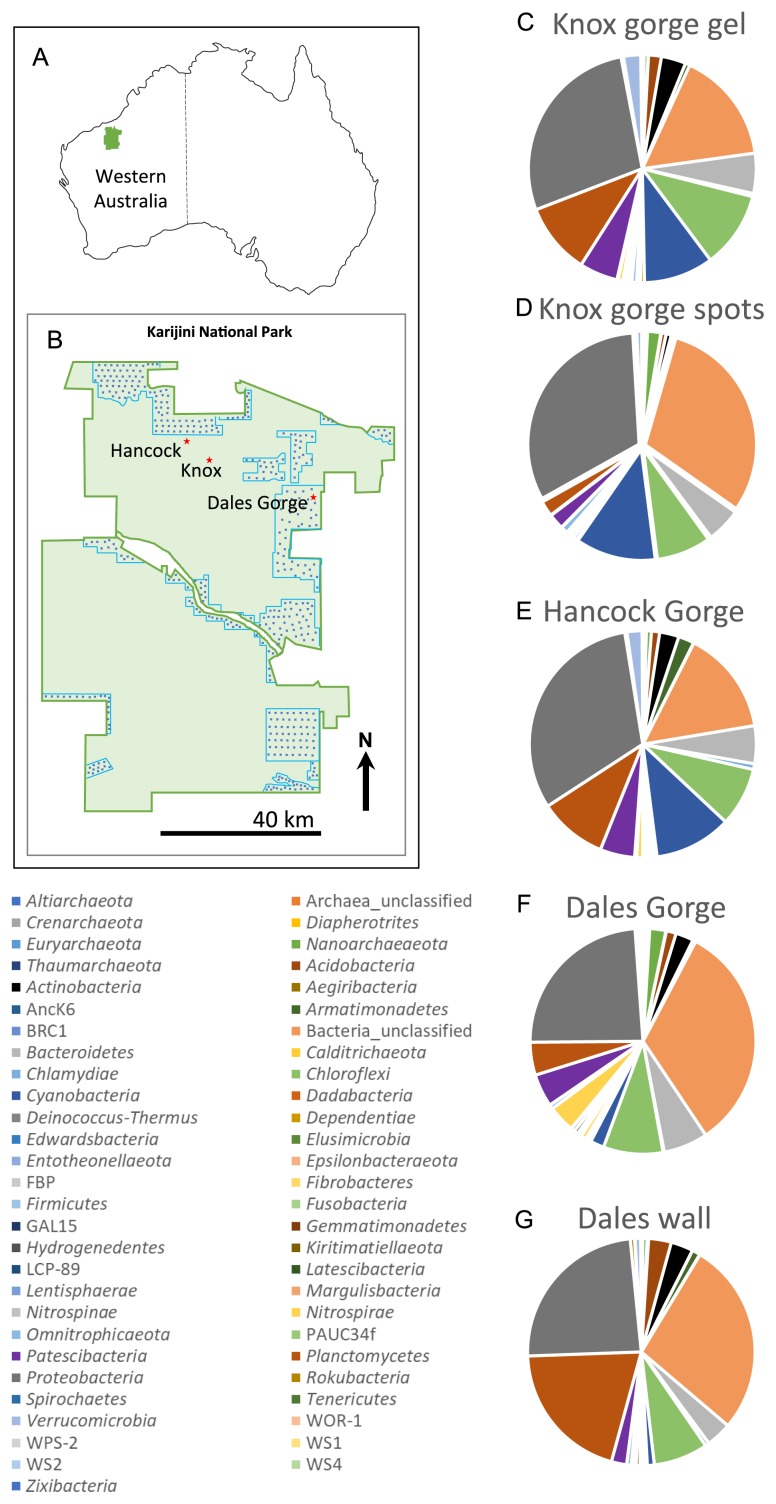
Karijini National Park in northwest Western Australia (A, boundary marked in green) is completely surrounded by active and pending mining tenements. Tenements that also include regions inside the national park boundary are marked with light blue lines and filled with blue dots (B). The locations of Hancock, Knox, and Dales Gorges are marked with red stars, indicating approximate sampling locations (B). The phylum level classification of unique 16S rRNA gene sequences collected from five iron precipitates at these locations in Karijini National Park are shown as pie charts (C to G).

**Fig. 2:**
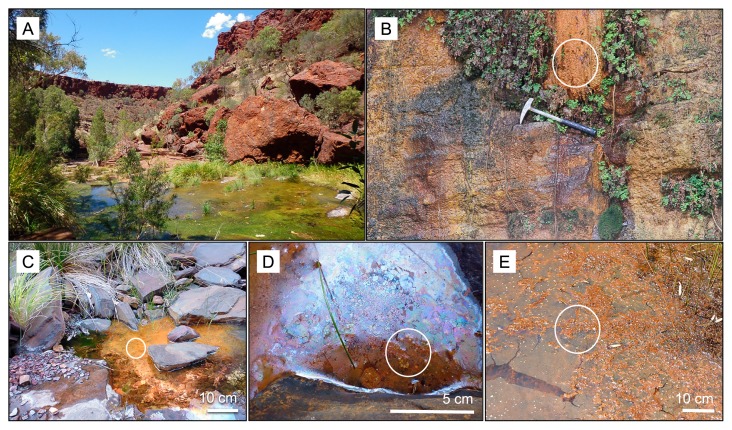
An overview of Dales Gorge (A) showing the highly weathered BIF environment that forms gorges in Karijini in the background and a pool of water in the foreground. This scenery was typical of all three gorges sampled, with water bodies of varying sizes along the lengths of each gorge and water seeping from the gorge walls (B) in various places. The locations at which iron features and water samples were collected have been highlighted with white circles for iron precipitates on plants growing down the wall at Dales Gorge (B); an iron mat in Knox Gorge (C) (note, a similar iron mat was sampled in a pool at Dales Gorge, photo not shown); iron-coated bubbles and sheen (D) from Knox Gorge (the same pool as in image C); and iron precipitates accumulating on plant material and forming as a surface sheen in a pond (E) at Hancock Gorge.

**Fig. 3:**
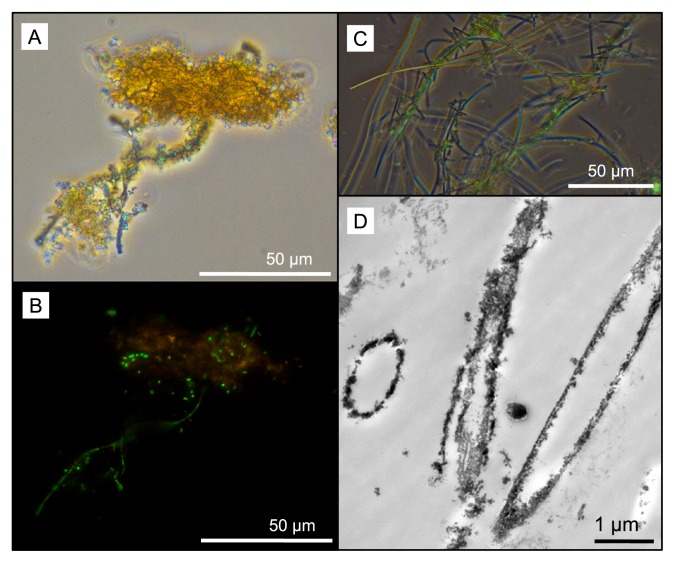
Phase (A) and UV (B) light microscopy of the same field of view of a sample of the Knox Gorge iron mat after staining with SYTO9^®^. SYTO9^®^ targets nucleic acids (green under UV). Scale bars are 50 μm. Overlay of phase and UV light microscopy images (C) of sheaths in the Dales Gorge iron mat after staining with SYTO9^®^, scale bar 50 μm. Transmission electron micrograph (D) showing a cross-section through a sheath, Dales Gorge iron mat, scale bar 1 μm, respectively. Note, electron density in this ultra-thin sectioned sample is the result of naturally immobilized metals; heavy metal fixatives to increase contrast between internal cell components and embedding resin were not used in TEM sample preparation.

When sequences were grouped at the phylum level, *Proteobacteria* or sequences not classifiable below the domain level dominated all samples. *Planctomycetes*, *Chloroflexi*, and *Cyanobacteria* were generally the next most abundant phyla (Fig. 1C–G). *Archaea* represented only 0.9 to 3.3% of all sequences per sample. A total of 187 of 1,595 OTUs were bacterial sequences not classifiable below the domain level and these comprised between 14.9 and 32.9% of sequences per sample (Fig. 1C–G). The structures of the microbial communities in each sample significantly differed (*P*<0.001, weighted and unweighted UniFrac tests). A principle coordinates analysis of differences in microbial communities may be explained by four principle coordinate axes and elements that correlated with variance along the two major axes including Mo, S, Ba, Ca, K, Mg, Na, Co, and Pb (Fig. 5).

The dominant OTU in the Dales Gorge iron mat (OTU 6, Fig. 6) was identical across the sequenced 16S rRNA gene region to *Sideroxydans lithotrophicus*, a well-described neutrophilic iron-oxidizing bacterium (13). Other OTUs that likely originated from iron-oxidizing species were identified in all samples. OTUs 8 and 14 demonstrated identity to members of the *Sphaerotilus-Leptothrix* group, which contains many heterotrophic iron- and/or manganese-depositing bacteria (44). OTU 101 from plants growing on the wall at Dales Gorge showed identity (though only 96% across the region sequenced) to an organism reported to be capable of iron oxidation, *Pedomicrobium australicum* (18). OTUs that demonstrated >97% identity across the sequenced region to organisms capable of iron reduction included OTU 2, *Anaeromyxobacter dehalogens* (20), and OTU 12 *Geobacter lovleyi* (42) present in all samples, except iron precipitates on plants growing on the wall at Dales Gorge.

Both of the Knox Gorge samples were dominated by cyanobacteria (OTU 1, Fig. 6), which was also the main cyanobacteria present in the Dales Gorge pool. The dominant OTU in Hancock Gorge (OTU 10, Fig. 6) was not closely related to a named isolate in the public domain; however, predominant OTUs were cyanobacteria from diverse lineages (OTUs 20, 21, and 26, Fig. 6). The sample collected from plants on the wall at Dales Gorge was also dominated by OTUs without close cultured relatives (OTUs 5 and 25, Fig. 6); however, the 3^rd^ and 5^th^ most abundant OTUs in that sample showed 97% 16S rRNA gene identity to ammonia oxidizers (OTUs 55 and 60 respectively, Fig. 6) (28, 45). Major OTUs (*i.e.*, the 10 most abundant per sample) that demonstrated >97% 16S rRNA gene identity to various aerobic heterotrophic bacteria were detected in all samples and were often shared across samples (OTUs 18, 4, 57, 3, and 46, Fig. 6). Major OTUs from lineages without close cultured relatives were common in all samples (highlighted in blue, Fig. 6).

## Discussion

**Fig. 4:**
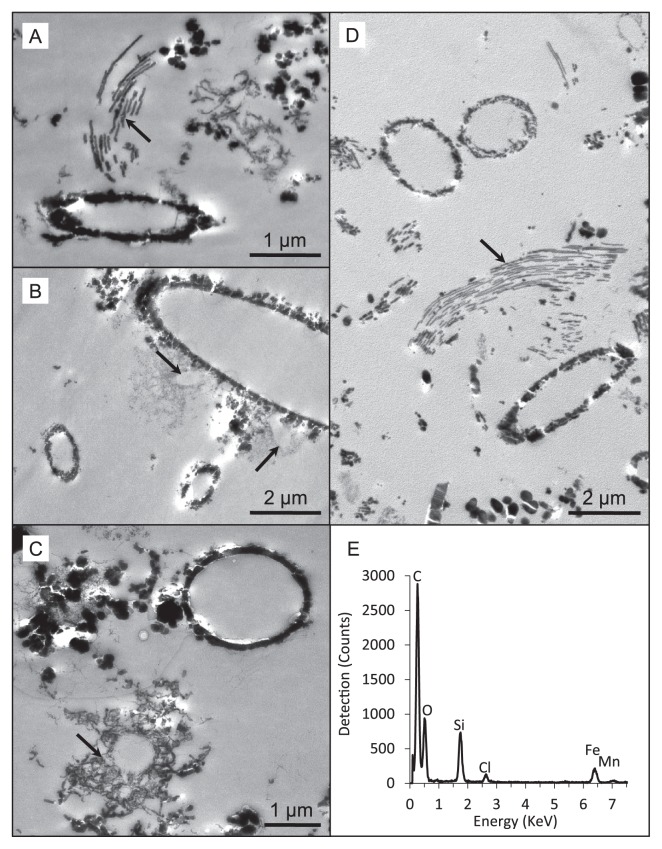
Transmission electron micrographs of Knox Gorge iron spots and iron mats (A, B, C) and the iron precipitate from plants growing down the wall at Dales Gorge (D). Features referred to in-text are marked by arrows as follows: iron oxidizer stalks in A and D; very fine accumulation of metals around cells in B in contrast to the denser precipitation around the larger cell they are near; metal accumulation amongst exopolymeric substances in C. Electron density in these ultra-thin sectioned samples is the result of naturally immobilized metals; heavy metal fixatives to increase contrast between internal cell components and embedding resin were not used in sample preparation. Scale bars are 1 μm (A, C) or 2 μm (B, D). Representative EDS spectrum (E) of a cell that was coated in electron dense minerals, showing primarily Fe, C, O, and Si with a minor contribution from Mn.

**Fig. 5:**
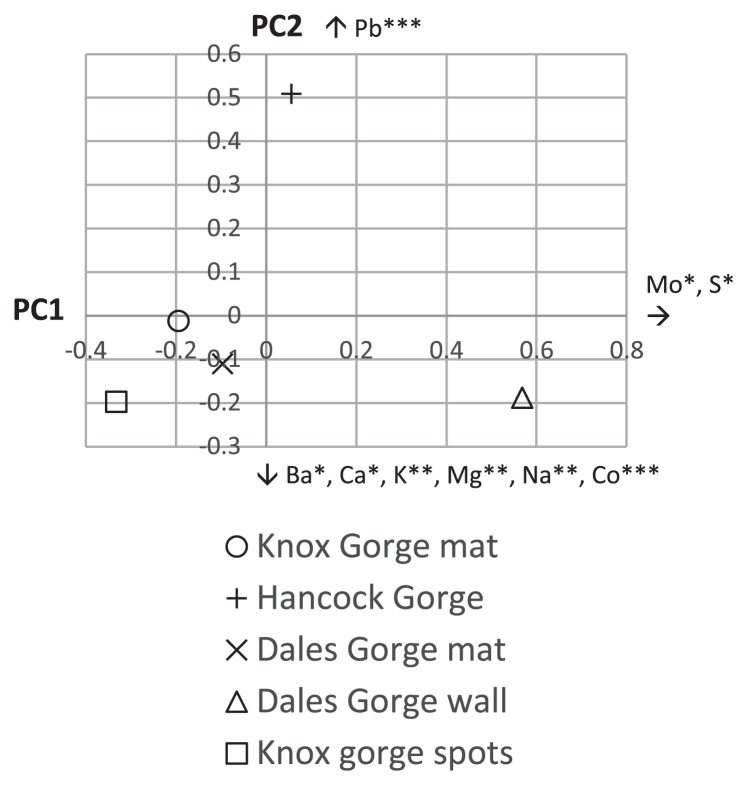
Principle coordinates analysis clustering of microbial communities of five iron features sampled at Karijini National Park after singletons in the entire dataset were removed. The analysis was performed on distances calculated using the Jaccard coefficient and OTUs clustered at a distance of ≤0.03. The first and second axes represent 40.0 and 29.0%, respectively, of the variance in the Jaccard distances between the communities. Elements that correlated with variance along the axes (assessed by Pearson’s correlation coefficient) are indicated with an arrow showing the direction in which they contribute and the significance of the association represented as *=*P*<0.05, **=*P*<0.01, ***=*P*<0.001.

The microscopic and molecular evidence presented here supports a role for microorganisms in the present-day precipitation of iron oxides within Karijini National Park. Iron precipitation on cell membranes results from passive metal adsorption and precipitation on functional groups (for an overview on the underlying mechanisms, see [27, 39]). In these Karijini samples, we noted different mineral assemblages around cells even within the same sample (*e.g.*, fine and dense accumulation, Fig. 4B; cell surface precipitation vs precipitation in a matrix of extrapolymeric substances, Fig. 4C), suggesting that differences in cell envelope properties or perhaps microbial activity contribute to different mineral structures. Oxygenic microbes potentially promoted denser mineral assemblage due to oxygen production at their cell surface, resulting in rapid iron oxide precipitation. The detection of iron-oxidizing microbial lineages in our samples also suggests a contribution from direct enzymatic iron oxidation. Features interpreted as stalks produced by some neutrophilic iron oxidizers (compare Fig. 4A and D to Fig. 1B in Krepski *et al.* [30]; Fig. 1C in Suzuki *et al.* [43]; Fig. 2E and F in Emerson *et al.* [12]) were evident. Known stalk-producing iron-oxidizing bacteria (*e.g.*, some members of *Gallionellaceae* and *Zetaproteobacteria*) were not detected using molecular methods, and, thus, potentially originate from novel iron oxidizers *e.g.*, see Krepski *et al.* (30). Structures reminiscent of sheaths produced by members of the *Leptothrix-Sphaerotilus* group (40) were also observed, although *Leptothrix* was not a major OTU (70^th^ most abundant) in one of the samples (Dales Gorge pond) in which they were readily identifiable using LM. Many of the sheaths in that sample appeared to be empty, which is common for *Leptothrix* (11), and may explain the lower number of 16S rRNA gene sequences detected than the multitude of sheath structures.

All known neutrophilic oxygen-dependent lithotrophic iron oxidizers from freshwater systems are *Betaproteobacteria* (22). The samples examined in the present study had a lower proportion of *Betaproteobacteria* at 4–13% of all sequences than other freshwater iron mats, wetlands, or groundwater seep communities, at between 26 and 75% of all clones as *Betaproteobacteria* (2, 3, 17, 46). Within *Betaproteobacteria*, *Gallionellaceae* represented ≤2.3% of sequences in Karijini samples, in contrast to up to 25% of all clones in other studies (2), while *Burkholderiaceae* (into which *Lepthothrix-Sphaerotilus* is classified) represented up to 6.0% of all clones; however, *Leptothrix* is sometimes not detected by molecular methods in other natural iron-rich systems (3, 46).

Similar to other iron-rich natural environments (2, 3, 46), iron-reducing bacteria were also present in four of the five samples—none were detected in the iron precipitates coating plants on the wall at Dales Gorge. The two OTUs (OTU 2 and 12) from potential iron-reducing bacteria were shared between the four samples, and *A. dehalogens* (OTU 2) was a major OTU in each of these samples (2^nd^, 3^rd^, 10^th^, and 11^th^ most abundant in Dales pool, Knox mat, Knox spot, and Hancock Gorge samples, respectively). The highest concentrations of soluble iron were measured in the Dales Gorge pool water sample in which *A. dehalogens* was the predominant OTU, while the lowest soluble iron concentration (7–28-fold lower than other samples) was measured in the water dripping out of the wall at Dales Gorge in which iron oxides were precipitating on plants, but known iron-reducing bacteria were not detected. Based on aqueous pH values (pH 5–6), the soluble iron measured in these samples was interpreted as ferrous iron (41).

Iron-reducing and iron-oxidizing bacteria have the ability to exist in close proximity to each other in the aerobic zone and contribute to the ongoing cycling of iron even though iron reduction is an anaerobic process (17, 35). In environmental studies, the co-existence of iron-oxidizing and -reducing bacteria is often indicative of the coupling of microbial iron oxidation and reduction processes (2, 3, 46). Laboratory studies have demonstrated that it is the activity of iron-reducing bacteria at a microscale anoxic-oxic interface, rather than the activity of iron-oxidizing bacteria, that drives iron oxide precipitation at circumneutral pH in an overall oxidizing environment (35). The presence of abundant and shared iron-reducing bacteria in four of the five Karijini iron precipitates sampled in the present study is consistent with the hypothesis proposed by Roden *et al.* (35) from observations based on microcosm experiments. Iron oxide accumulated in the natural environment sampled for the present study, rather than remaining trapped in a ferrous-ferric cycle between iron-oxidizing and-reducing bacteria (35). Therefore, iron-reducing bacteria may exist in close association with aerobic heterotrophs or other oxygen-consuming bacteria in their immediate vicinity to maintain micro-anoxic niches for iron reduction, while iron-oxidizing bacteria exist further away at a higher oxygen concentration in the same sample. The iron-oxidizing bacterium that was also detected in four of the five Karijini samples was a *Leptothrix* OTU, and *Leptothrix* has been shown to prefer higher oxygen concentrations (10) than the microaerophilic neutrophilic iron oxidizers that may keep iron trapped in a ferrous-ferric cycle in association with iron reducers (35).

It is important to note that our results differ from recent findings in Brazil. Parker *et al.* (34) linked microbial iron reduction in the highly weathered BIF systems in Brazil to cave formation, *i.e.*, the overall broad scale dissolution rather than net formation of iron oxides. This requires further investigation and is potentially related to oxygen gradients and the presence of organic matter to drive long-term anaerobic conditions for net mineral dissolution that may lead to cave formation, whereas the niches sampled in Karijini National Park for the present study were aerobic overall and appeared to be exposed to ongoing active oxygen generation based on the abundance of the phototrophic oxygenic microbial lineages detected. This may be a critical factor in the overall net precipitation of iron oxides that leads to the ongoing formation and stabilization of iron duricrusts in these ecosystems.

The iron precipitate on plants growing on the wall at Dales Gorge was broadly the most different microbiologically of the five samples, with only one major OTU potentially linked to iron cycling (OTU 101) and no phototrophic microbial lineages. The low soluble Fe in this sample (Table 1) suggests that the active biogeochemical cycling of iron (particularly iron reduction) may be limited despite the abundant amounts of visible iron precipitates. This was the only sample that was not recovered from a sitting water body, which may be a large driver for the difference in the microbial community structure from those in other samples. The mineralized stalks evident in this sample (Fig. 4D) may originate from novel iron oxidizers. Stalk formation is an important trait for neutrophilic iron oxidizers and is one of the identifying features of this metabolism in nature (30). However, without supporting functional gene or cultivation data, it is impossible to speculate which of the OTUs identified in this sample may be linked to stalk formation and iron oxidation. Seven of the top 10 OTUs in this sample originate from species for which there is presently no close cultured relative; thus, apart from an indication of microbial ammonia oxidation, there is little else that may be inferred about microbial functionality in this sample at this stage.

**Fig. 6:**
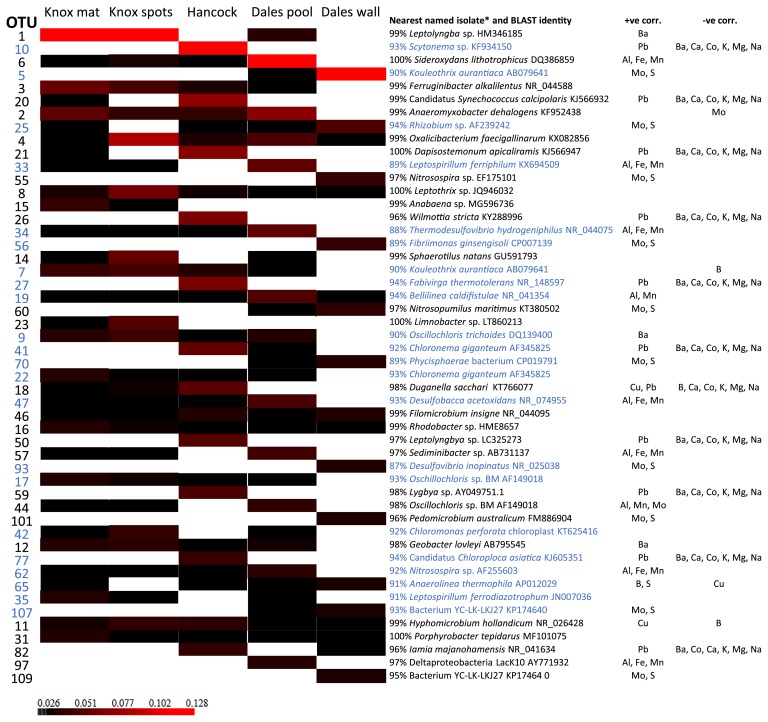
Heatmap analysis of 16S rRNA gene sequences from five iron precipitates in Karijini National Park, showing the most abundant OTUs for each sample. The analysis was performed for OTUs clustered at a distance of ≤0.03. The scale bar indicates the relative abundance of each OTU within a sample group from black (least abundant) to red (most abundant) and with white representing undetected OTUs. The nearest named isolate in the public domain and its accession number (preference given to published studies) is given for each OTU. OTUs with no close cultured representative in the public domain (*i.e.*, <95% 16S rRNA gene identity) are marked in light blue. The elements are listed beside each OTU in cases in which there was a significant (*P*<0.05) Pearson’s correlation between OTUs and any geochemical data.

The abundance of microbial sequences from uncultivated or deep branching lineages in all samples in the present study is of interest (Fig. 6 and 1C–G). Many of these sequences were only classified to the class or order level and the absence of close cultured relatives indicates that it is impossible to infer anything about the role of these organisms in the environment based on phylogeny alone. We examined correlation coefficients between OTUs and the geochemical data collected at each site and found correlations with some elements (Fig. 6). These OTUs from uncultivated lineages that correlated positively with Al, Fe, and/or Mn must not be overlooked as potential contributors to metal cycling in Karijini and included OTUs 33, 34, 19, 47, 44, and 62 (Fig. 6). In the future, with cultivation and/or genomic information on relatives to these uncultivated and often phylogenetically deep-branching microorganisms, their possible function in nature will become clearer.

In terms of the ecological or biotechnological significance of the present results, a replication of the natural iron oxide formation processes observed in the field may be used as a strategy to promote the formation of fresh iron oxides that contribute to the cementation of iron-rich duricrusts *e.g.*, in post-mining restoration attempts to reform surface cangas. Overall processes were consistent in several samples collected from across the park. Iron reduction by *A. dehalogens* and *G. lovleyi* was fundamental in driving the reductive dissolution of iron oxide minerals, and these iron reducers may exist in close partnership with general aerobic heterotrophs unique to each sample. Phototrophy was critical for the ongoing generation of oxygen to drive aerobic conditions, which is where this system may differ from cave formation processes (and net iron oxide dissolution) in Brazilian canga areas. Heterotrophic iron-depositing *Leptothrix* species, which grow at sufficiently high concentrations of oxygen to be distant from iron reducers, may have contributed to overall net iron oxide precipitation. Based on these commonalities, the provision of nutrients (N, P) and an appropriate electron donor for iron reducers (*e.g.*, lactate or acetate) and a carbon source for *Leptothrix* and heterotrophs (*e.g.*, glucose or peptone) under mining remediation conditions may be sufficient to drive the reduction and subsequent re-oxidation of abundant iron oxides in, for example, crushed BIF or waste iron oxides from mining under water-saturated conditions. Under natural sunlight and with abundant water and nutrients, naturally present phototrophic oxygenic organisms thrive, providing the conditions that favor fresh iron oxide precipitation at the oxic-anoxic interface. With dehydration (natural evaporation under the arid conditions around Karijini), freshly formed iron oxides transform to more crystalline phases (6). Thus, the stimulation of basic microbial processes and subsequent dehydration of the system may be a platform for the reformation of iron-cemented duricrusts from crushed BIF or promotion of the stabilization of iron oxide waste piles.

## Figures and Tables

**Table 1:** Major cations (ppb) and pH of water samples in which distinctive iron precipitates were forming in Karijini National Park.

	pH	Al	B	Ba	Ca	Co	Cu	Fe	K	Mg	Mn	Mo	Na	Pb	S
**Dales Gorge small pool ~ 0.3×0.2 m.** **Iron mat**	5.75	50	1,427	51	44,858	2	5	2,202	12,974	36,829	235	<DL	72,711	<DL	6,069
**Dales Gorge wall seepage.** **Iron precipitate on wall plants.**	5.75	<DL	1,391	52	41,132	2	3	81	12,638	42,074	5	2	80,789	<DL	12,250
**Knox Gorge small pool ~ 1×0.2 m.** **Iron mats and iron spots.**	5.50	<DL	363	69	28,145	2	12	581	10,510	32,581	96	1	67,369	<DL	2,805
**Hancock Gorge pond ~ 3×1 m.** **Iron precipitate on pond plants.**	5.00	<DL	119	31	8,490	1	15	1,481	6,611	16,689	49	1	45,991	8	132

Elements that were below the detection limit in all samples were as follows: As, Cd, Cr, Ni, P, Se, and Zn. Detection limits are listed in Methods.
